# Sepsis Alert Systems, Mortality, and Adherence in Emergency Departments: A Systematic Review and Meta-Analysis

**DOI:** 10.1001/jamanetworkopen.2024.22823

**Published:** 2024-07-22

**Authors:** Hyung-Jun Kim, Ryoung-Eun Ko, Sung Yoon Lim, Sunghoon Park, Gee Young Suh, Yeon Joo Lee

**Affiliations:** 1 Division of Pulmonary and Critical Care Medicine, Department of Internal Medicine, Seoul National University Bundang Hospital, Seongnam, Republic of Korea; 2 Department of Internal Medicine, Seoul National University College of Medicine, Seoul, Republic of Korea; 3 Department of Critical Care Medicine, Samsung Medical Center, Sungkyunkwan University School of Medicine, Seoul, Republic of Korea; 4 Department of Pulmonary, Allergy and Critical Care Medicine, Hallym University Sacred Heart Hospital, Anyang, Republic of Korea

## Abstract

**Importance:**

Early detection and management of sepsis are crucial for patient survival. Emergency departments (EDs) play a key role in sepsis management but face challenges in timely response due to high patient volumes. Sepsis alert systems are proposed to expedite diagnosis and treatment initiation per the Surviving Sepsis Campaign guidelines.

**Objective:**

To review and analyze the association of sepsis alert systems in EDs with patient outcomes.

**Data Sources:**

A thorough search was conducted in PubMed, EMBASE, Web of Science, and the Cochrane Library from January 1, 2004, to November 19, 2023.

**Study Selection:**

Studies that evaluated sepsis alert systems specifically designed for adult ED patients were evaluated. Inclusion criteria focused on peer-reviewed, full-text articles in English that reported on mortality, ICU admissions, hospital stay duration, and sepsis management adherence. Exclusion criteria included studies that lacked a control group or quantitative reports.

**Data Extraction and Synthesis:**

The review followed the Preferred Reporting Items for Systematic Reviews and Meta-analyses (PRISMA) reporting guideline. Two independent reviewers conducted the data extraction using a standardized form. Any disagreements were resolved through discussion. The data were synthesized using a random-effects model due to the expected heterogeneity among the included studies.

**Main Outcomes and Measures:**

Key outcomes included mortality, intensive care unit admissions, hospital stay duration, and adherence to the sepsis bundle.

**Results:**

Of 3281 initially identified studies, 22 (0.67%) met inclusion criteria, encompassing 19 580 patients. Sepsis alert systems were associated with reduced mortality risk (risk ratio [RR], 0.81; 95% CI, 0.71 to 0.91) and length of hospital stay (standardized mean difference [SMD], −0.15; 95% CI, −0.20 to −0.11). These systems were also associated with better adherence to sepsis bundle elements, notably in terms of shorter time to fluid administration (SMD, −0.42; 95% CI, −0.52 to −0.32), blood culture (SMD, −0.31; 95% CI, −0.40 to −0.21), antibiotic administration (SMD, −0.34; 95% CI, −0.39 to −0.29), and lactate measurement (SMD, −0.15; 95% CI, −0.22 to −0.08). Electronic alerts were particularly associated with reduced mortality (RR, 0.78; 95% CI, 0.67 to 0.92) and adherence with blood culture guidelines (RR, 1.14; 95% CI, 1.03 to 1.27).

**Conclusions and Relevance:**

These findings suggest that sepsis alert systems in EDs were associated with better patient outcomes along with better adherence to sepsis management protocols. These systems hold promise for enhancing ED responses to sepsis, potentially leading to better patient outcomes.

## Key Points

**Question:**

Are sepsis alert systems, including electronic clinical decision supports and manual notifications, associated with mortality and adherence to the Surviving Sepsis Campaign sepsis bundle for initial treatment of patients in the emergency department?

**Findings:**

In this systematic review and meta-analysis including 22 studies of 19 580 patients, sepsis alert systems were associated with lower mortality, a shorter hospital stay, and improved sepsis-bundle adherence, notably in terms of shorter time to fluid administration, blood culture, antibiotic administration, and lactate measurement. Electronic alerts were particularly associated with reducing mortality and time to blood culture.

**Meaning:**

This systematic review and meta-analysis found that sepsis alert systems were associated with better outcomes and adherence with sepsis management in emergency departments.

## Teaser

This systematic review and meta-analysis assesses the associations of sepsis alert systems in emergency departments with mortality and adherence to sepsis management guidelines.

## Introduction

Sepsis is a major global health problem associated with high mortality and morbidity rates among hospitalized patients.^1,2^ A recent global analysis estimated that sepsis accounted for 11 million deaths in 2017, accounting for 19.7% of all deaths worldwide.^3^ To improve the quality of sepsis management, the Surviving Sepsis Campaign (SSC) developed an evidence-based bundle comprising 5 elements to be implemented within the first hours of sepsis recognition.^4^ These elements include fluid resuscitation with 30 mL/kg of crystalloid, blood culture before antibiotic administration, broad-spectrum antibiotic therapy, lactate measurement, and vasopressor support for refractory hypotension. Adherence to the sepsis bundle has demonstrated improved clinical outcomes in patients,^5,6,7^ despite the varying quality of evidence supporting some of the elements.

Patients with sepsis who visit the emergency department (ED) exhibit high rates of hospitalization (70%) and mortality (10%). The ED is the main point of entry for hospital admission in this population.^8,9^ Timely diagnosis and treatment of sepsis are critical, as delays can significantly increase the risk of mortality and morbidity.^5,10^ However, ED staff face challenges in promptly identifying and effectively managing sepsis due to the frequent overcrowding of patients in need of urgent care.^11^ To address these challenges, sepsis alert systems have emerged as potential solutions to facilitate the early recognition of sepsis in the ED. Several studies have investigated the role of sepsis alert systems in improving the early identification of sepsis in patients in the ED. A 2020 systematic review examined the diagnostic accuracy of sepsis alert systems and found that they had acceptable sensitivity for identifying patients with sepsis.^12^ However, the clinical impact of sepsis alert systems on patient outcomes, including mortality rates and timelines of sepsis management in the ED, remains unclear.

Therefore, we conducted a systematic review and meta-analysis to assess the association of sepsis alert systems with mortality and adherence to the SSC sepsis bundle for the initial management of patients with sepsis in the ED. By synthesizing the available evidence, we aimed to provide insights into the potential benefits of sepsis alert systems in improving clinical outcomes and optimizing sepsis management in the challenging ED environment.

## Methods

### Search Strategy

For this systematic review and meta-analysis, the study protocol was registered in PROSPERO (record identifier: 341106). We searched PubMed, EMBASE, Web of Science, and the Cochrane Library with keywords, such as *emergency department*, and *sepsis alert system*. Because the SSC was launched in 2004, we only considered studies published after that date.^13^ The search was conducted on November 19, 2023, using a structural search plan (eAppendix in Supplement 1). We manually searched the references of pertinent review publications for additional papers.We followed the Meta-analysis of Observational Studies in Epidemiology (MOOSE) reporting guideline and Preferred Reporting Items for Systematic Reviews and Meta-analyses (PRISMA) reporting guideline in accordance with the appropriate checklists.^14,15,16^

### Study Selection and Definition of Outcomes

All study designs were included. Sepsis alert systems were defined as interventions using mechanisms to facilitate early sepsis identification and treatment. Titles and abstracts were reviewed to identify studies that qualified for full-text evaluation. We selected only peer-reviewed English full-text articles with eligible target patients (adult patients with sepsis in the ED), the appropriate intervention (alert systems for sepsis), comparators (absent alert system), and outcomes that were presented in numbers. We evaluated the outcomes that may be associated with sepsis alert systems, such as death, intensive care unit (ICU) admission, hospital length of stay (LOS), and adherence to the SSC sepsis bundle (intravenous [IV] fluid administration, acquisition of blood culture, antibiotic administration, and lactate measurement).

### Data Extraction

We collected data from each study, including author names, study duration, country, study design, number of patients, and target patients. Each study’s detailed description included the criteria of the alert system, the method of alert delivery, and subsequent interventions. As the definitions of each outcome (death, ICU admission, hospital LOS, and adherence to each sepsis bundle) varied among the studies, the definitions in each study were also described.

Information was categorized based on type. The input variables for the categorical variables were organized in a 2-by-2 table. For continuous variables, means and SDs were calculated, as suggested by the Cochrane Handbook.^17^

### Statistical Analysis

Using forest plots and a random-effects model, we investigated the baseline characteristics and the association of each variable with outcomes. *I*^2^ statistics were used to determine the heterogeneity levels. Pooled risk ratios (RRs) were calculated for categorical variables. Owing to differences in scales, standardized mean differences (SMDs) were calculated for continuous variables. SMDs describe the effect size relative to the variability observed in each study, facilitating comparisons across studies with different scales or units. For example, if the SD is 1 hour, an SMD of −0.5 implies treatment occurs approximately 30 minutes sooner. The 95% CIs for each pooled value were calculated. The statistical analyses in this meta-analysis were guided by a predefined significance level of *P* < .05, using 2-sided tests. According to the recommended risk of bias in nonrandomized studies of interventions (ROBINSON-I) protocol,^18^ the risk of bias in each study was assessed for the following 7 domains: bias due to confounding factors, selection of participants, classification of interventions, deviations from intended interventions, missing data, measurement of outcomes, and selection of reported results. Egger regression test was used to assess publication bias.^19^

We planned a subgroup analysis based on the type of alert system (electronic alert vs nonelectronic alert) to assess the differential associations of sepsis alert systems. Electronic alerts referred to automated alerts, such as pop-up windows in electronic health records (EHRs), while nonelectronic alerts referred to manual interventions, like broadcasting notifications within the ED. Due to significant heterogeneity in the time to implement each bundle approach, we performed additional subgroup analyses including only studies with a low risk of bias.

Two independent reviewers (H.-J.K. and R.-E.K.) performed study screening, data extraction, and assessment of quality and risk of bias, and a consensus was reached through group discussion. All statistical analyses were performed using Stata software version 17 (StataCorp). Data were analyzed from November 19, 2023, to May 3, 2024.

## Results

### Search Findings and Study Characteristics

**Figure 1.**
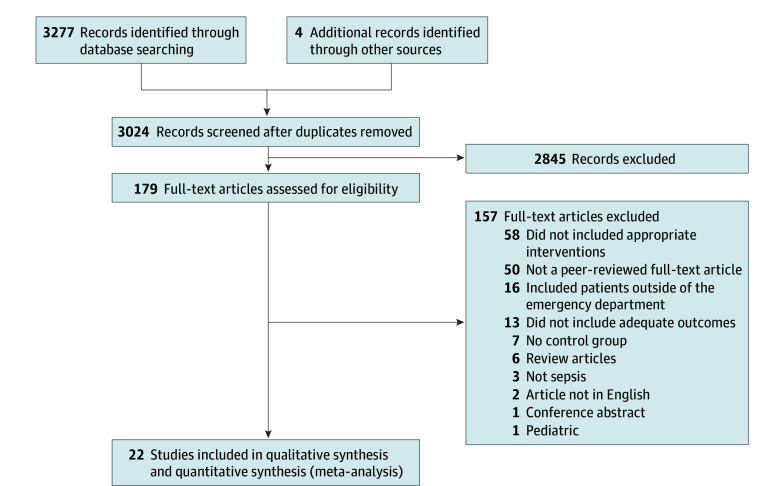
Flowchart of the Study Selection Process

The initial search yielded 3281 studies. After duplicate removal and screening the titles and abstracts, 179 articles were fully reviewed. After excluding 157 irrelevant studies, we analyzed 22 studies (Figure 1).^20,21,22,23,24,25,26,27,28,29,30,31,32,33,34,35,36,37,38,39,40,41^

The 22 studies included 19 580 patients, with 10 344 (52.8%) in the treatment groups (sepsis alert system implementation) and 9236 (47.2%) in the control groups (no sepsis alert system implementation). Most studies were from the United States (11 studies),^20,22,24,27,29,30,31,33,36,38,39^ followed by Australia,^23,41^ Sweden,^26,37^ and Canada^21,28^ (2 studies each). Most studies were observational,^20,21,22,23,24,25,26,27,28,29,30,31,32,33,34,35,36,37,39,40,41^ with only 1 prospective randomized clinical trial.^38^ All studies focused on adult patients admitted to the ED with suspected sepsis, with some variations in patient characteristics (Table 1). Details of the alert system criteria, method of alert delivery, predefined subsequent interventions, and definitions of each outcome are provided in the eTable in Supplement 2. In 12 studies (54.5%),^20,22,24,25,27,28,29,32,33,34,38,39^ the alert was delivered via electronic means. In the remaining 10 studies,^21,23,26,30,31,35,36,37,40,41^ the alert was delivered using conventional methods.

**Table 1.** Characteristics of Studies Included in the Meta-Analysis

Source	Study duration	Country	Study design	Patients, No. treatment/control	Target patients
Berger et al,^20^ 2010	2009-2010	US	Prospective pre/post quasi-experimental study	2893/2903	≥2 SIRS criteria + suspected infection by physician^a^
Patocka et al,^21^ 2014	2015, 2008	Canada	Retrospective observational study	170/185	Suspected sepsis or septic shock
Hayden et al,^22^ 2016	2012-2013, 2013-2014	US	Retrospective observational study	130/108	Sepsis, severe sepsis, and septic shock based on *ICD-9* codes and medical records reviews
Idrees et al,^23^ 2016	2013, 2014	Australia	Retrospective observational study	45/55	Adult patients fulfilling severe sepsis or septic shock criteria admitted to the ICU from the ED^b^
Narayanan et al,^24^ 2016	2012, 2013	US	Retrospective observational study	103/111	Severe sepsis or septic shock (*ICD-9* code and clinical definition per Surviving Sepsis Guideline)
Arabi et al,^25^ 2017	2011-2013	Saudi Arabia	Prospective and retrospective implementation study	195/436	Sepsis: ≥2 SIRS + organ dysfunction^c^ secondary to documented or suspected infection^a^; septic shock, defined as sepsis with persistent hypotension after fluid resuscitation ≥20 mL/kg of crystalloid
Rosenqvist et al,^26^ 2017	2010, 2012, 2014	Sweden	Retrospective observational study	152/69	Patients with both fever and fulfilling the vital signs criteria
Austrian et al,^27^ 2018	2013-2015	US	Retrospective observational study	1306/838	Discharge diagnosis as sepsis or septic shock (*ICD-9*)
McDonald et al,^28^ 2018	2014-2015	Canada	Retrospective observational study	270/346	Patients diagnosed with sepsis, defined as SIRS with suspected or confirmed source of infection
Shah et al,^29^ 2018	2012-2013, 2015	US	Retrospective observational study	57/58	Patients diagnosed with sepsis or septic shock based on *ICD-9* codes and medical records reviews
Borrelli et al,^30^ 2019	2016	US	Retrospective observational study	20/43	Severe sepsis (≥2 SIRS + suspected infection + evidence of organ dysfunction [lactate >18.02 mg/dL, acute mental status change, hypotension, and oxygen saturation <90%])^a^ or septic shock^d^
Moore et al,^31^ 2019	Not stated	US	Retrospective observational study	202/110	Positive sepsis screenings or diagnosis
Song et al,^32^ 2019	2016-2017, 2017-2019	South Korea	Retrospective observational study	315/316	Meeting the diagnostic criteria for sepsis (Sepsis-3), age ≥18 y; qSOFA ≥2 on ED arrival
Delawder et al,^33^ 2020	2017	US	Retrospective observational study	107/107	Sepsis, severe sepsis, and septic shock
Honeyford et al,^34^ 2020	2016-2018	United Kingdom	Retrospective observational study	2695/1927	Patients with positive alerts of the infection response or evidence of organ dysfunction.
Petit et al,^35^ 2020	2017-2018	France	Prospective pre-post quasiexperimental study	350/328	Clinical signs suggestive of bacterial infection
Threatt et al,^36^ 2020	2017-2019	US	Observational study (not specified as retrospective)	145/165	Final diagnosis as severe sepsis and septic shock
Rosenqvist et al,^37^ 2020	2015, 2017	Sweden	Retrospective observational study	558/508	Patients with the highest RETTS priority group (red) in ED^e^
Tarabichi et al,^38^ 2022	2016-2019	US	Prospective randomized clinical trial	285/313	Patients who triggered the EWS flag^f^
Troncoso Jr et al,^39^ 2023	2018-2019	US	Retrospective observational study	109/98	Patients within the emergency medical system with suspected sepsis
Schinkel et al,^40^ 2023	2019-2022	The Netherlands	Prospective pre-post quasiexperimental study	133/132	Adult patients (age ≥18 y) who presented to the ED with a suspected infection and a Modified Early Warning Score ≥3 during their stay
Roman et al,^41^ 2023	2015-2018	Australia	Prospective and retrospective implementation study	104/80	Admitted to the emergency and trauma center with suspected sepsis and admitted to the ICU

Abbreviations: ED, emergency department; EWS, early warning score; *ICD-9*, *International Classification of Diseases, Ninth Revision*; ICU, intensive care unit; qSOFA, quick sequential organ failure assessment; RETTS, rapid emergency triage and treatment system; SIRS, systemic inflammatory response syndrome.

SI conversion factor: To convert lactate to millimoles per liter, multiply by 0.111.

^a^ SIRS criteria are body temperature greater than 38 °C or less than 36 °C; heart rate greater than 90 beats/min; respiratory rate greater than 20 breaths/min; white blood cell count greater than 12 000/μL or less than 4000/μL (to convert to ×10^9^/L, multiply by 0.001).

^b^ Definitions of severe sepsis and septic shock are sepsis-induced hypotension, lactate greater than upper limits of reference range, urine output less than 0.5 mL/kg/h for more than 2 hours despite adequate fluid resuscitation, acute lung injury with arterial partial pressure of oxygen (Pao_2_) or fraction of inspired oxygen (Fio_2_) less than 250 in the absence of pneumonia as infection source, acute lung injury with Pao_2_ or Fio_2_ less than 200 in the absence of pneumonia as infection source, creatinine greater than 2.0 mg/dL (to convert to micromoles per liter, multiply by 76.25), total bilirubin greater than 2 mg/dL (to convert to micromoles per liter, multiply by 17.104), platelet count less than 100 ×10^3^/μL (to convert to ×10^9^/L, multiply by 1), coagulopathy (international normalized ratio >1.5), evidence of hypotension or hypoperfusion (indicated as systolic blood pressure <90 mm Hg after 20 mL/kg crystalloid bolus or lactate ≥36.04 mg/dL).

^c^ Defined as systolic blood pressure 86 to 90 mm Hg with intravenous fluids or less than 86 mm Hg regardless of fluids; or blood oxygen saturation of 85% to 90% with supplemental oxygen or less than 85% without oxygen; or lactate greater than 18.02 mg/dL.

^d^ Severe sepsis and persistent hypotension or end organ dysfunction despite fluid resuscitation or vasopressors.

^e^ At least 1 of the following: respiratory rate less than 8 or greater than 30 breaths/min, oxygen saturation as measured by pulse oximetry less than 90% despite supplementary oxygen, heart rate greater than 130 beats per minute (>160 beats per minute if irregular), altered mentation (Swedish Reaction Level Scale 85 > 4 or Glasgow Coma Scale <9), ongoing seizure, petechiae, obstructed airway, stridor, lactate level 31.53 md/dL or greater.

^f^ Predeveloped logistic regression model composed of demographic data, vital signs, laboratory results, orders, and comorbidities.

### Effect of Sepsis Alerts on Patient Outcomes

**Figure 2.**
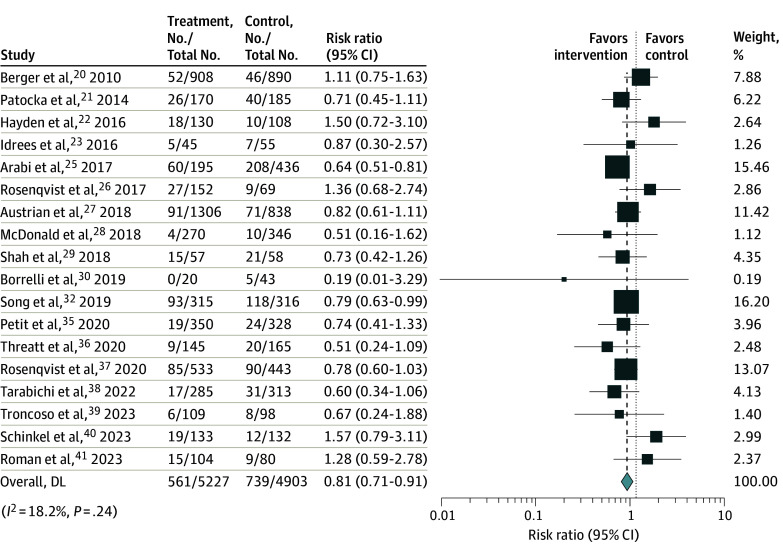
Association of Sepsis Alert Systems With Mortality of Patients in the Emergency Department

There were 18 studies^20,21,22,23,25,26,27,28,29,30,32,35,36,37,38,39,40,41^ that evaluated mortality as the outcome. The definitions of death in each study were in-hospital (11 studies^20,21,22,23,25,27,28,30,35,39,41^), 28-day (4 studies^26,37,38,40^), and 30-day (2 studies^29,32^) mortality; 1 study^36^ did not state the definition. The overall mortality rate was 14% (95% CI, 11% to 18%). Implementation of the sepsis alert system was associated with decreased mortality risk (RR, 0.81; 95% CI, 0.71 to 0.91) (Figure 2).

We found 9 studies^22,26,27,28,29,35,37,38,40^ that evaluated ICU admission rates. ICU admission was described as occurring at any time (7 studies^22,26,27,29,37,38,40^), within 24 hours (1 study^28^), or within 48 hours (1 study^35^). The overall rate of admission to ICUs was 25% (95% CI, 16% to 35%). The overall risk of ICU admission was not associated with the presence of a sepsis alert system (RR, 0.94; 95% CI, 0.79 to 1.13) (eFigure 1 in Supplement 1). According to 15 studies^22,23,25,26,27,29,30,31,32,35,36,37,38,40,41^ that evaluated the hospital LOS, the hospital LOS was shorter in the sepsis alert system group than the no alert system control group (SMD, −0.15; 95% CI, −0.20 to −0.11) (eFigure 2 in Supplement 1).

### Association of Sepsis Alerts With Sepsis Bundle Adherence

Studies that explicitly specified time limits were included in the analysis of the association of sepsis alerts with sepsis bundle adherence. Time zero had different definitions in each study; it was defined as the point of ED admission (9 studies^22,25,26,27,32,36,38,39,41^), the point of triage (6 studies^20,21,23,28,35,37^), the point at which the first set of vital signs was obtained (2 studies^29,30^), the time of the alert alarm (1 study^34^), the point of recognition of sepsis (1 study^33^), the point of the earliest EHR annotation consistent with all elements of severe sepsis or septic shock (1 study^31^), or when the patient met specific criteria (2 systemic inflammatory response syndrome criteria: evidence of end-organ dysfunction and a suspected infection [1 study^24^]). The remaining study^40^ did not specify the definition of time zero (Supplement 2).

As a whole, application of the sepsis alert system was associated with a higher rate of adherence to each element of the sepsis bundle (Table 2). The subgroup analyses (electronic vs nonelectronic alert) yielded results similar to those of the main analysis methods for most outcomes, except for mortality and blood culture (Table 2). The electronic alert system was associated with reduced mortality (RR, 0.78; 95% CI, 0.67 to 0.92) and adherence to blood culture guidelines (RR, 1.14; 95% CI, 1.03 to 1.27), whereas the nonelectronic alert systems showed no notable difference.

**Table 2.** Association of Electronic Alert Systems With Adherence to Sepsis Bundle Protocols

Outcome	Studies, No.	Treatment vs control estimate (95% CI)	*I*^2^ for heterogeneity, %	Egger *P* value
**Overall**				
Mortality	18	0.71 (0.71 to 0.91)^a^^,^^b^	18.2	.53
Admission to ICU	9	0.94 (0.79 to 1.13)^b^	64.8	.08
Length of hospital stay	15	−0.15 (−0.20 to −0.11)^a^^,^^c^	54.6	.74
Adherence to a timely bundle approach				
Fluid administration	4	1.11 (1.01 to 1.23)^a^^,^^b^	92.3	.89
Blood culture	9	1.18 (1.07 to 1.30)^a^^,^^b^	92.4	.39
Antibiotics administration	9	1.52 (1.30 to 1.77)^a^^,^^b^	87.6	.04
Lactate measurement	5	1.81 (1.36 to 2.40)^a^^,^^b^	98.5	.01
**Electronic alert group**				
Mortality	9	0.78 (0.67 to 0.92)^a^^,^^b^	22.9	.73
Admission to ICU	5	0.90 (0.73 to 1.11)^b^	74.7	.23
Length of hospital stay	6	−0.18 (−0.24 to −0.12)^a^^,^^c^	46.5	.81
Adherence to a timely bundle approach				
Fluid administration	3	1.22 (1.01 to 1.47)^a^^,^^b^	91.4	.85
Blood culture	6	1.14 (1.03 to 1.27)^a^^,^^b^	94.0	.65
Antibiotics administration	5	1.24 (1.08 to 1.42)^a^^,^^b^	75.5	.34
Lactate measurement	4	1.77 (1.30 to 2.40)^a^^,^^b^	98.7	.02
**Nonelectronic alert group**				
Mortality	9	0.86 (0.68 to 1.07)^b^	19.3	.80
Admission to ICU	4	1.08 (0.77 to 1.50)^b^	28.1	.71
Length of hospital stay	9	−0.12 (−0.19 to −0.05)^a^^,^^c^	59.8	.81
Adherence to a timely bundle approach				
Fluid administration	1	1.52 (1.17 to 1.97)^a^^,^^b^	NA	NA
Blood culture	3	1.47 (0.94 to 2.31)^b^	90.6	.26
Antibiotics administration	4	2.30 (1.30 to 4.07)^a^^,^^b^	91.7	.06
Lactate measurement	1	1.95 (1.56 to 2.42)^a^^,^^b^	NA	NA

Abbreviations: ICU, intensive care unit; NA, not applicable.

^a^ Statistically significant.

^b^ Expressed as risk ratio.

^c^ Expressed as standardized mean difference.

**Figure 3.**
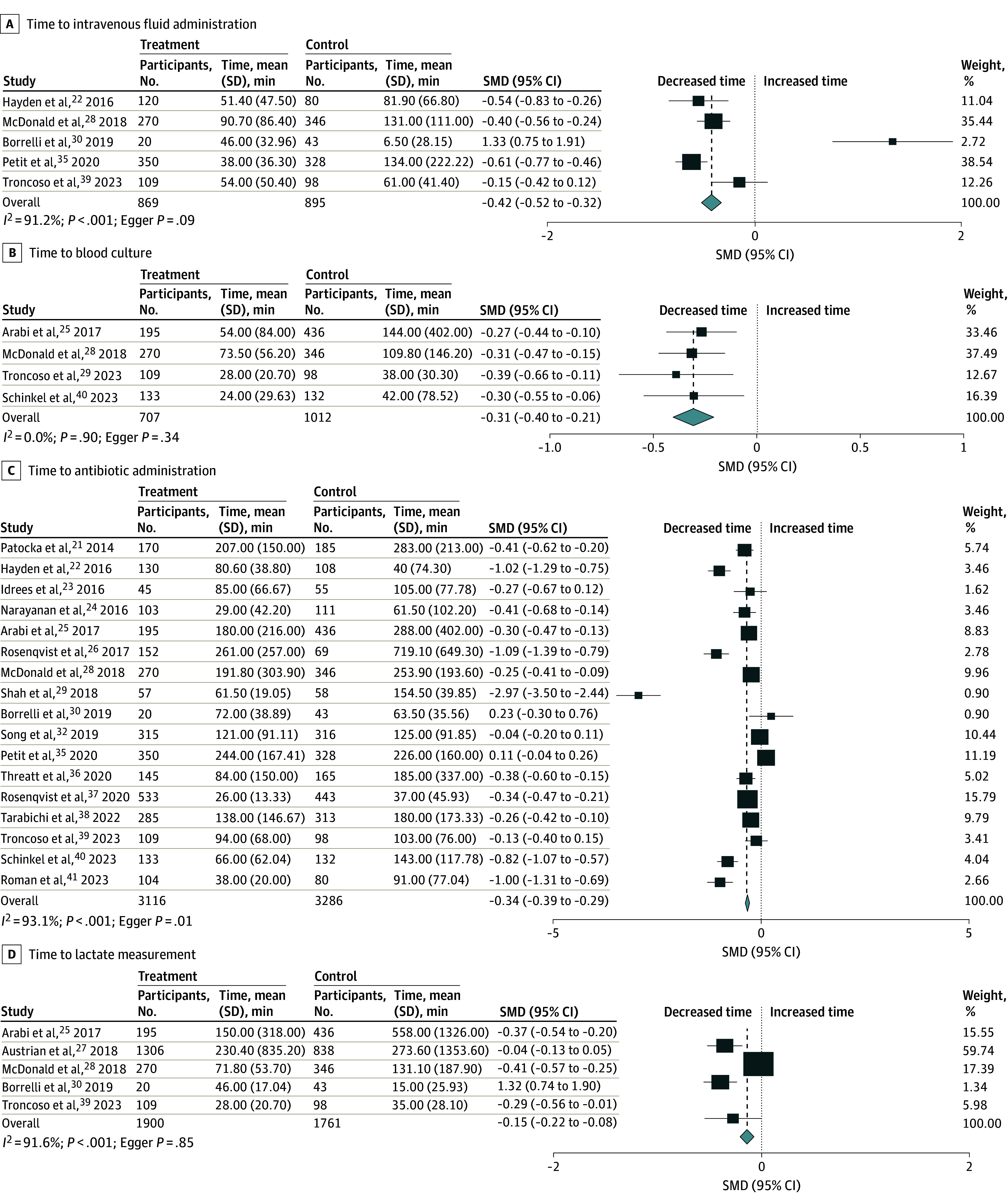
Association of Sepsis Alerts With the Time to Each Sepsis Bundle Event in the Emergency Department SMD indicates standardized mean difference.

The duration between time zero and each sepsis bundle step was also evaluated, including time to IV fluid administration (5 studies^22,28,30,35,39^), blood culture (4 studies^25,28,29,40^), antibiotic administration (17 studies^21,22,23,24,25,26,28,29,30,32,35,36,37,38,39,40,41^), and lactate measurement (5 studies^25,27,28,30,39^). The implementation of the sepsis alert system in the ED was associated with earlier IV fluid administration (SMD, −0.42; 95% CI, −0.52 to −0.32), blood culture (SMD, −0.31; 95% CI, −0.40 to −0.21), antibiotic administration (SMD, −0.34; 95% CI, −0.39 to −0.29), and lactate measurement (SMD, −0.15; 95% CI, −0.22 to −0.08) (Figure 3). We performed another subgroup analysis focusing on studies assessed as having a low risk of bias concerning the time to implement each bundle component (eFigure 3 in Supplement 1). Despite this, the results remained consistent.

### Assessment of Bias

With ROBINSON-I, 16 of the 22 studies had an overall low risk of bias.^21,24,25,26,28,29,30,31,32,34,35,36,37,38,39,40^ Four studies^20,22,27,41^ had moderate risk, and 2 studies^23,33^ had serious risks of overall bias (eFigure 4 in Supplement 1).

Publication bias was not observed for mortality rate (Egger intercept, 0.33; 95% CI, −0.77 to 1.44; *P* = .53) (eFigure 5 in Supplement 1), ICU admission (Egger intercept, 1.81; 95% CI, −0.28 to 3.91; *P* = .08), or hospital LOS (Egger intercept, 0.32; 95% CI, −1.68 to 2.32; *P* = .74). Regarding sepsis bundle steps, the time to antibiotic administration (Egger intercept, −5.98; 95% CI, −10.59 to −1.38; *P* = .01) and the proportion of lactate measurements in studies (Egger intercept, 10.71; 95% CI, 4.18 to 17.23; *P* = .01) revealed substantial publication bias.

## Discussion

This systematic review and meta-analysis aimed to assess the association of sepsis alert systems with mortality risk and adherence to the sepsis bundle for the initial management of patients with sepsis in the ED. The main findings of this study can be summarized as follows: (1) patient mortality risk was significantly associated with the presence of sepsis alert systems, (2) sepsis alert systems were associated with enhanced adherence to the sepsis bundle and a shorter time to delivery of each bundle element, and (3) the sepsis alert systems had more associations for the electronic alert group than for the nonelectronic alert group.

We found that the implementation of the sepsis alert system was significantly associated with a lower mortality risk compared with the control group. A meta-analysis of 36 studies by Zhang et al^42^ evaluated the effectiveness of the sepsis alert system in ED, general ward, and ICU settings. Zhang et al^42^ reported a significant reduction in mortality (29%) with sepsis alert systems compared with usual care. However, another meta-analysis of 16 studies by Joshi et al^43^ that evaluated the associations of sepsis alert systems in the ED and general wards found no significant association with mortality reduction. The conflicting results may be due to the heterogeneity of patients with sepsis targeted by these meta-analyses. In contrast, we included only ED settings to ensure a similar context for the recognition and management of sepsis. We found that the sepsis alert system was significantly associated with lower mortality and a shorter hospital LOS, which is consistent with the findings from Joshi et al.^43^

The implementation of the sepsis alert system was associated with a shorter time to intervention for all 4 elements of the sepsis bundle. Previous meta-analyses on this topic also attempted to evaluate adherence to elements of the sepsis bundle but were limited to the time to antibiotic administration. Joshi et al^43^ found no significant difference in antibiotic timing between groups with and without a sepsis alert system based on 5 studies. However, in another systematic review, Hwang et al^12^ reported that sepsis alert systems might reduce the time to antibiotics. Reducing the time interval from the recognition of sepsis to the implementation of the sepsis bundle is a crucial indicator of the quality of care in initial sepsis management.^7,44^ However, this measure is challenging due to the inconsistency in determining time zero.^12,42,43,45^ Hence, our study aimed to evaluate the association of sepsis alert systems with the time interval from the recognition of sepsis to the implementation of the sepsis bundle by focusing only on patients in the ED, which enabled a more precise definition of time zero. However, we acknowledge that the definition of time zero in the included studies was not uniform. A key objective for future research is to standardize the definition of time zero in patients with sepsis, especially in the general ward and ICU, as this would enable a more accurate analysis of sepsis outcomes across different settings. Despite substantial publication bias regarding the time to blood culture and time to lactate measurement, to our knowledge, our meta-analysis is the first to demonstrate that the sepsis alert system was associated with a shorter time interval from the recognition of sepsis to the implementation of all sepsis bundle components.

Our analysis found that the sepsis alert system had more associations within the electronic alert systems than the nonelectronic systems. In our study, the electronic alert group had a lower mortality, shorter hospital LOS, and significantly better adherence to each bundle component. However, the nonelectronic alert group showed no significant association with mortality or adherence to blood culture. The introduction of EHRs has enabled the application of electronic alerts as a sepsis alert system.^43,46^ Evidence has demonstrated the use of electronic alert systems across a spectrum of diseases.^47,48^ In high-volume, diverse patient scenarios, such as EDs, the need for distinct and efficient alert systems is crucial. The electronic alert system is integrated into the EHR and uses the information from the EHR to quickly alert clinicians to patients with sepsis, thereby improving their bundle adherence and reducing patient mortality rates.

However, careful consideration must be given to the potential for false-positive alarms, which vary significantly depending on the methods used. Our meta-analysis includes studies that reported a wide range of sensitivities for detecting sepsis.^27,35^ This variability underscores the persistent risk of false positives. Comprehensive research is essential to better understanding the impacts of sepsis alert systems, particularly focusing on the rate of false positives and their associated consequences, such as resource overutilization and the overprescription of antibiotics.

### Limitations

This study had some limitations. First, the definition of sepsis was not uniform across the studies included in our systematic review and meta-analysis. This may be due to the retrospective observational design of most studies, which relied on criteria, such as systemic inflammatory response syndrome and quick sequential organ failure assessments, to define sepsis. Moreover, these variations may reflect the evolution of the definition of sepsis over time. However, we confirmed that the degree of heterogeneity was not a concern among studies regarding mortality as an outcome. Second, blinding was difficult due to the nature of the interventions. Most studies were not randomized and prone to confounding factors. Third, most management protocols included steps for sepsis management after sepsis alert system interventions. Consequently, clinical outcomes might be influenced by these interventions. To maximize the effectiveness of sepsis alert systems, it is crucial to consider the entire sepsis care process, from early recognition to timely treatment. Further research is needed to identify the specific interventions that should be implemented in conjunction with sepsis alert systems to optimize patient outcomes. Fourth, despite examining all bundle components, we could not demonstrate the results of vasopressor use. Only 2 studies assessed the use of vasopressors; however, they only reported whether vasopressors were used, without providing any information on their application in response to hypotension during or after fluid resuscitation. Fifth, while our study presents findings regarding the mean time to implementation of sepsis bundle components, it lacks comprehensive data on the proportion of patients whose care fully adhered to all aspects of the sepsis bundle within the critical first hour of recognition. Due to the diverse definitions used in the included studies, our analysis could not definitively determine complete adherence within the specified time frame.

## Conclusions

This systematic review and meta-analysis demonstrated the significant association of implementing sepsis alert systems in the ED with patient outcomes. These findings indicate that sepsis alert systems were not only associated with reduced mortality risk but also higher and quicker adherence to the sepsis bundle. These beneficial outcomes were more frequently observed in the electronic alert systems. The results of this study highlight the potential of sepsis alert systems as valuable tools for improving the outcomes of adult patients with sepsis in the ED, thus emphasizing the importance of their widespread implementation and integration into clinical practice. Given the significant heterogeneity of the studies, careful interpretation is required, and future research with more controlled environments is needed.

## Notes

Supplement 1. **eAppendix.** Detailed Search Strategy for the Systematic Review **eFigure 1.** Association of Sepsis Alert System in the Emergency Department With Admission Rates to the Intensive Care Unit **eFigure 2.** Association of Sepsis Alert System in the Emergency Department With Length of Hospital Stay **eFigure 3.** Subgroup Analysis of Studies with Low Risk of Bias on the Time to Sepsis Bundle Implementation **eFigure 4.** Risk of Bias Traffic Light Plots for Each Study According to ROBINS-I **eFigure 5.** Funnel Plot Analysis of Publication Bias for Association of Alert Systems in the Emergency Department With Patient Mortality

Supplement 2. **eTable.** Details of the Studies Included in Analysis

Supplement 3.
Data Sharing Statement
