# Supplementary material for: Sepsis Alert Systems, Mortality, and Adherence in Emergency Departments: A Systematic Review and Meta-Analysis
Source: JAMA Netw Open. 2024 Jul 22;7(7):e2422823. doi: 10.1001/jamanetworkopen.2024.22823 (PMC11265133; doi:10.1001/jamanetworkopen.2024.22823)
Supplement: Supplement 1. — eAppendix. Detailed Search Strategy for the Systematic Review eFigure 1. Association of Sepsis Alert System in the Emergency Department With Admission Rates to the Intensive Care Unit eFigure 2. Association of Sepsis Alert System in the Emergency Department With Length of Hospital Stay eFigure 3. Subgroup Analysis of Studies with Low Risk of Bias on the Time to Sepsis Bundle Implementation eFigure 4. Risk of Bias Traffic Light Plots for Each Study According to ROBINS-I eFigure 5. Funnel Plot Analysis of Publication Bias for Association of Alert Systems in the Emergency Department With Patient Mortality [file jamanetwopen-e2422823-s001.pdf]

Supplemental Online Content

Kim H, Ko R, Lim SY, Park S, Suh GY, Lee YJ. Sepsis alert systems, mortality, and adherence in emergency departments. *JAMA Netw Open*. 2024;7(7):e2422823.
doi:10.1001/jamanetworkopen.2024.22823

eAppendix. Detailed Search Strategy for the Systematic Review

eFigure 1. Association of Sepsis Alert System in the Emergency Department With Admission Rates to the Intensive Care Unit

eFigure 2. Association of Sepsis Alert System in the Emergency Department With Length of Hospital Stay

eFigure 3. Subgroup Analysis of Studies with Low Risk of Bias on the Time to Sepsis Bundle Implementation

eFigure 4. Risk of Bias Traffic Light Plots for Each Study According to ROBINS-I

eFigure 5. Funnel Plot Analysis of Publication Bias for Association of Alert Systems in the Emergency Department With Patient Mortality

This supplemental material has been provided by the authors to give readers additional information about their work.

© 2024 Kim H et al. *JAMA Network Open.*

eAppendix. Detailed search strategy for the systematic review

PubMed

#1. Emergency room

"Emergency Service, Hospital"[Mesh] OR "Evidence-Based Emergency Medicine"[Mesh] OR "Resuscitation"[Mesh] OR "Emergency Medicine"[Mesh] OR emergency room*[tiab] OR emergency department*[tiab] OR emergency medicine*[tiab] OR emergency service*[tiab]

#2. Sepsis

"Sepsis"[Mesh] OR "Systemic Inflammatory Response Syndrome"[Mesh] OR "Shock, Septic"[Mesh] OR sepsis[tiab] OR septic shock[tiab]

#3. Alert/screening system

"Triage"[Mesh] OR "Early Warning Score"[Mesh] OR "Severity of Illness Index"[Mesh] OR "Medical Order Entry Systems"[Mesh] OR "Decision Support Systems, Clinical"[Mesh] OR alert[title] OR alert system*[tiab] OR triage[title] OR triage system*[tiab] OR warning score*[tiab] OR screening system*[tiab] OR surveillance system*[tiab] OR decision support system*[tiab] OR order entry system*[tiab] OR notification system*[tiab]

#4. Children

"child"[MeSH] OR child*[title]

#5. Preclinical

"Drug Evaluation, Preclinical"[Mesh] OR "In Vitro Techniques"[Mesh] OR "Animal Experimentation"[Mesh] OR "Models, Animal"[Mesh] OR "Disease Models, Animal"[Mesh] OR "Zoology"[Mesh] OR "veterinary" [Subheading] OR "Veterinary Medicine"[Mesh] OR “animal”[title] OR “in vitro”[title] OR “preclinical”[title] OR “pre-clinical”[title]

#6. Review

"Review" [Publication Type] OR "Systematic Review" [Publication Type] "Meta-Analysis as Topic"[Mesh] OR “systematic review”[title] OR meta-analysis[title] OR “meta analysis”[title]

(#1 AND #2 AND #3) NOT (#4 OR #5 OR #6)

(("emergency service, hospital"[MeSH Terms] OR "Evidence-Based Emergency Medicine"[MeSH Terms] OR "Resuscitation"[MeSH Terms] OR "Emergency Medicine"[MeSH Terms] OR "emergency room*"[Title/Abstract] OR "emergency department*"[Title/Abstract] OR "emergency medicine*"[Title/Abstract] OR "emergency service*"[Title/Abstract]) AND ("Sepsis"[MeSH Terms] OR "Systemic Inflammatory Response Syndrome"[MeSH Terms] OR "shock, septic"[MeSH Terms] OR "Sepsis"[Title/Abstract] OR "septic shock"[Title/Abstract]) AND ("Triage"[MeSH Terms] OR "Early Warning Score"[MeSH Terms] OR "Severity of Illness Index"[MeSH Terms] OR "Medical Order Entry Systems"[MeSH Terms] OR "decision support systems, clinical"[MeSH Terms] OR "alert"[Title] OR "alert system*"[Title/Abstract] OR "Triage"[Title] OR "triage system*"[Title/Abstract] OR "warning score*"[Title/Abstract] OR "screening system*"[Title/Abstract] OR "surveillance system*"[Title/Abstract] OR "decision support system*"[Title/Abstract] OR "order entry system*"[Title/Abstract] OR "notification system*"[Title/Abstract])) NOT ("child"[MeSH Terms] OR "child*"[Title] OR ("drug evaluation, preclinical"[MeSH Terms] OR "In Vitro Techniques"[MeSH Terms] OR

© 2024 Kim H et al. *JAMA Network Open.*

"Animal Experimentation"[MeSH Terms] OR "models, animal"[MeSH Terms] OR "disease models, animal"[MeSH Terms] OR "Zoology"[MeSH Terms] OR "veterinary"[MeSH Subheading] OR "Veterinary Medicine"[MeSH Terms] OR "animal"[Title] OR "in vitro"[Title] OR "preclinical"[Title] OR "pre-clinical"[Title]) OR ((("Review"[Publication Type] OR "Systematic Review"[Publication Type]) AND "Meta-Analysis as Topic"[MeSH Terms]) OR "Systematic Review"[Title] OR "meta-analysis"[Title] OR "meta-analysis"[Title]))

EMBASE

#1. Emergency room

'emergency health service'/exp OR 'emergency treatment'/exp OR 'resuscitation'/exp OR 'emergency medicine'/exp OR ‘emergency room*’:ab,ti OR ‘emergency department*’:ab,ti OR ‘emergency medicine*’:ab,ti OR ‘emergency service*’:ab,ti

#2. Sepsis

'sepsis'/exp OR 'systemic inflammatory response syndrome'/exp OR 'septic shock'/exp OR sepsis:ab,ti OR ‘septic shock’:ab,ti

#3. Alert/screening system

'patient triage'/exp OR 'early warning score'/exp OR 'disease severity assessment'/exp OR 'physician order entry system'/exp OR 'decision support system'/exp OR alert:ab,ti OR alert system*:ab,ti OR triage:ti OR ‘triage system*’:ab,ti OR ‘warning score*’:ab,ti OR ‘screening system*’:ab,ti OR ‘surveillance system*’:ab,ti OR ‘decision support system*’:ab,ti OR ‘order entry system*’:ab,ti OR ‘notification system*’:ab,ti

#4. Children

‘child’/exp OR child*:ti

#5. Preclinical

'preclinical study'/exp OR 'animal experiment'/exp OR 'in vitro study'/exp OR 'animal model'/exp OR 'zoology'/exp OR 'veterinary medicine'/exp OR animal:ti OR ‘in vitro’:ti OR preclinical:ti OR pre-clinical:ti

#6. Review

'review'/exp OR 'systematic review'/exp OR 'systematic review (topic)'/exp OR 'meta analysis (topic)'/exp OR 'meta analysis'/exp OR ‘systematic review’:ti OR meta-analysis:ti OR ‘meta analysis’:ti

(#1 AND #2 AND #3) NOT (#4 OR #5 OR #6)

Cochrane library

#1. Emergency room

[mh “Emergency Service, Hospital”] OR [mh "Evidence-Based Emergency Medicine"] OR [mh Resuscitation]

© 2024 Kim H et al. *JAMA Network Open.*

OR [mh “Emergency Medicine”] OR (emergency room*):ti,ab,kw OR (emergency department*):ti,ab,kw OR (emergency medicine*):ti,ab,kw OR (emergency service*):ti,ab,kw

#2. Sepsis

[mh Sepsis] OR [mh "Systemic Inflammatory Response Syndrome"] OR [mh "Shock, Septic"] OR sepsis:ti,ab,kw OR (septic shock):ti,ab,kw

#3. Alert/screening system

[mh Triage] OR [mh "Early Warning Score"] OR [mh "Severity of Illness Index"] OR [mh "Medical Order Entry Systems"] OR [mh "Decision Support Systems, Clinical"] OR alert:ti OR (alert system*):ti,ab,kw OR triage:ti OR (triage system*):ti,ab,kw OR (warning score*):ti,ab,kw OR (screening system*):ti,ab,kw OR (surveillance system*):ti,ab,kw OR (decision support system*):ti,ab,kw OR (order entry system*):ti,ab,kw OR (notification system*):ti,ab,kw

#4. Children

[mh child] OR child:ti

#5. Preclinical

[mh "Drug Evaluation, Preclinical"] OR [mh "In Vitro Techniques"] OR [mh "Animal Experimentation"] OR [mh "Models, Animal"] OR [mh "Disease Models, Animal"] OR [mh Zoology] OR [mh "Veterinary Medicine"] OR animal:ti OR “in vitro”:ti OR preclinical:ti OR pre-clinical:ti

#6. Review

[mh Review] OR [mh "Systematic Review"] OR [mh "Meta-Analysis as Topic"] OR “systematic review”:ti OR “meta-analysis”:ti

(#1 AND #2 AND #3) NOT (#4 OR #5 OR #6)

Web of science (core collection)

#1. Emergency room

TS = ("Emergency Service, Hospital" OR "Evidence-Based Emergency Medicine" OR "Resuscitation" OR "Emergency Medicine" OR (emergency room*) OR (emergency department*) OR (emergency medicine*) OR (emergency service*))

#2. Sepsis

TS = (Sepsis OR "Systemic Inflammatory Response Syndrome" OR "Shock, Septic" OR “septic shock”)

#3. Alert/screening system

(TS = (Triage OR "Early Warning Score" OR "Severity of Illness Index" OR "Medical Order Entry Systems" OR

"Decision Support Systems, Clinical" OR (alert system*) OR (warning score*) OR (screening system*) OR (surveillance system*) OR (decision support system*) OR (order entry system*) OR (notification system*))) OR (TI = (alert))

#4. Children

TI = (child*)

#5. Preclinical

(TS = ("Drug Evaluation, Preclinical" OR "In Vitro Techniques" OR "Animal Experimentation" OR "Models, Animal" OR "Disease Models, Animal" OR "Zoology" OR "veterinary" OR "Veterinary Medicine")) OR (TI = (animal OR “in vitro” OR preclinical OR “pre-clinical”))

#6. Review

TI = (“systematic review” OR meta-analysis OR “meta analysis”)

(#1 AND #2 AND #3) NOT (#4 OR #5 OR #6)

© 2024 Kim H et al. *JAMA Network Open.*

eFigure 1. Association of Sepsis Alert System in the Emergency Department with Admission Rates to the Intensive Care Unit

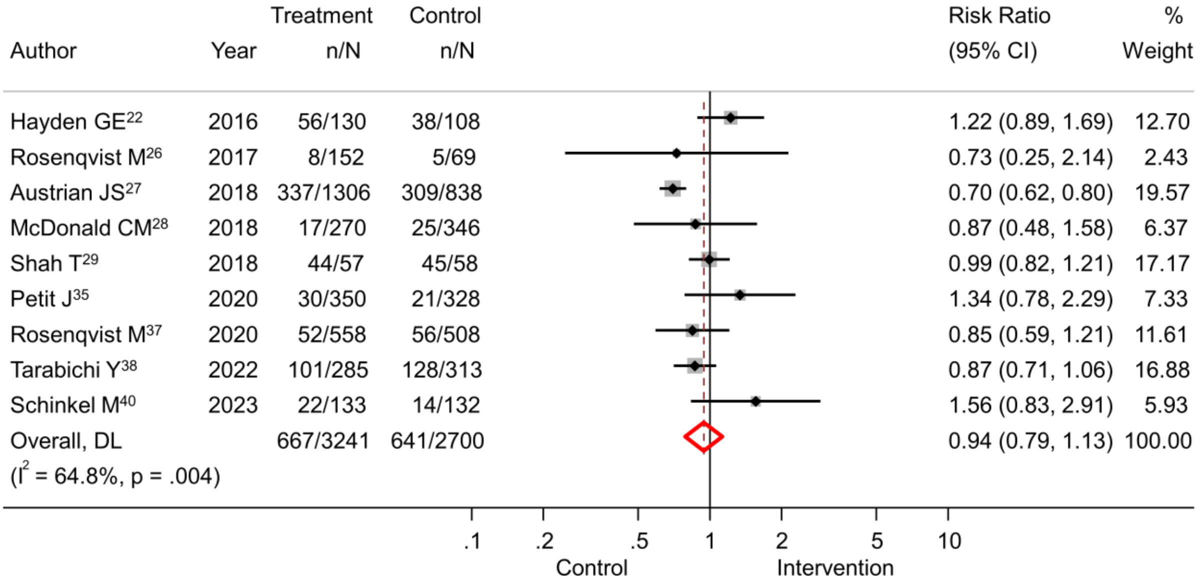

Egger's regression test did not detect significant publication bias (p = .08).

© 2024 Kim H et al. *JAMA Network Open.*

eFigure 2. Association of Sepsis Alert System in the Emergency Department with Length of Hospital Stay

Author	Year	Treatment N	Treatment Mean (SD)	Control N	Control Mean (SD)	SMD (95% CI)	% Weight
Hayden GE[22]	2016	130	9.00 (10.10)	108	8.20 (9.20)	0.08 (-0.17, 0.34)	3.22
Idrees M[23]	2016	45	7.00 (4.44)	55	8.00 (8.89)	-0.14 (-0.53, 0.26)	1.35
Arabi YM[25]	2017	195	8.60 (8.30)	436	13.30 (17.40)	-0.31 (-0.48, -0.14)	7.29
Rosenqvist M[26]	2017	152	13.30 (10.40)	69	16.60 (12.40)	-0.30 (-0.58, -0.01)	2.57
Austrian JS[27]	2018	1306	8.60 (7.90)	838	10.10 (10.10)	-0.17 (-0.26, -0.08)	27.82
Shah T[29]	2018	57	7.50 (2.90)	58	9.00 (3.48)	-0.47 (-0.84, -0.10)	1.53
Borrelli G[30]	2019	20	5.00 (2.22)	43	8.00 (5.19)	-0.67 (-1.21, -0.13)	0.71
Moore WR[31]	2019	202	213.00 (76.03)	110	220.00 (90.94)	-0.09 (-0.32, 0.15)	3.89
Song J[32]	2019	315	9.00 (8.15)	316	10.00 (8.89)	-0.12 (-0.27, 0.04)	8.61
Petit J[35]	2020	350	6.00 (5.20)	328	7.00 (6.70)	-0.17 (-0.32, -0.02)	9.23
Threatt DL[36]	2020	145	9.17 (8.97)	165	9.15 (10.77)	0.00 (-0.22, 0.23)	4.22
Rosenqvist M[37]	2020	558	6.00 (4.44)	508	7.00 (5.19)	-0.21 (-0.33, -0.09)	14.46
Tarabichi Y[38]	2022	285	3.20 (3.78)	313	4.00 (4.15)	-0.20 (-0.36, -0.04)	8.12
Schinkel M[40]	2023	133	5.00 (5.19)	132	4.00 (5.19)	0.19 (-0.05, 0.43)	3.61
Roman CP[41]	2023	269	11.23 (10.03)	80	9.77 (9.91)	0.15 (-0.10, 0.40)	3.37
Overall, IV (I^2 = 54.6%, p = .006)		4162		3559		-0.15 (-0.20, -0.11)	100.00

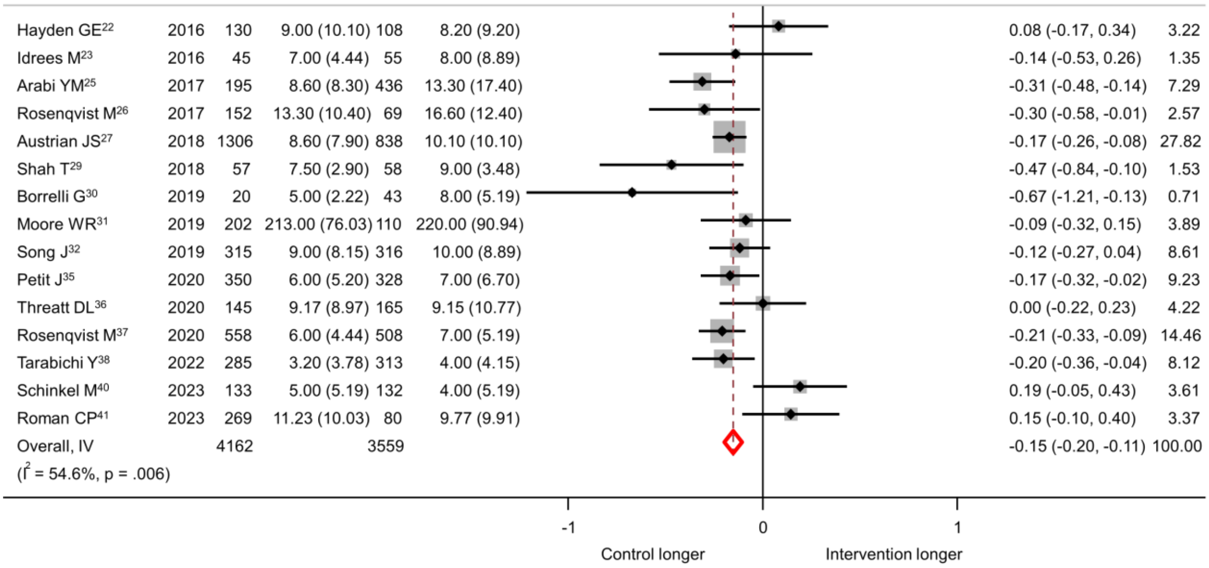

Egger's regression test did not detect significant publication bias (*p* =.74).

© 2024 Kim H et al. *JAMA Network Open.*

eFigure 3. Subgroup Analysis of Studies with Low Risk of Bias on the Time to Sepsis Bundle Implementation

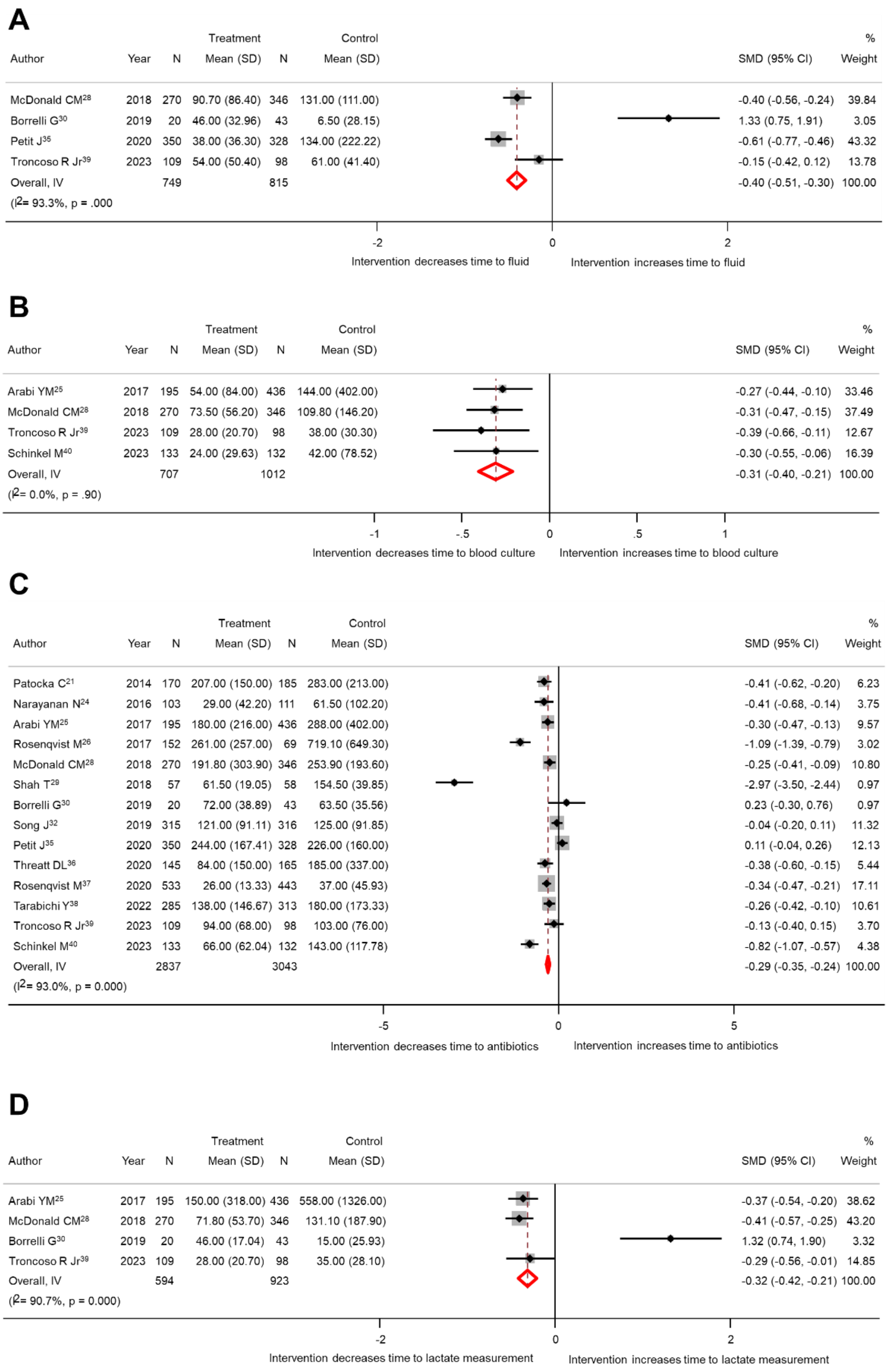

Each forest plot refers to (A) time to intravenous fluid administration, (B) time to blood culture, (C) time to antibiotic administration, and (D) time to lactate measurement. Abbreviations: SD, standard deviation; SMD, standardized mean difference; CI, confidence interval.

© 2024 Kim H et al. *JAMA Network Open.*

eFigure 4. Risk of Bias Traffic Light Plots for Each Study According to ROBINS-I

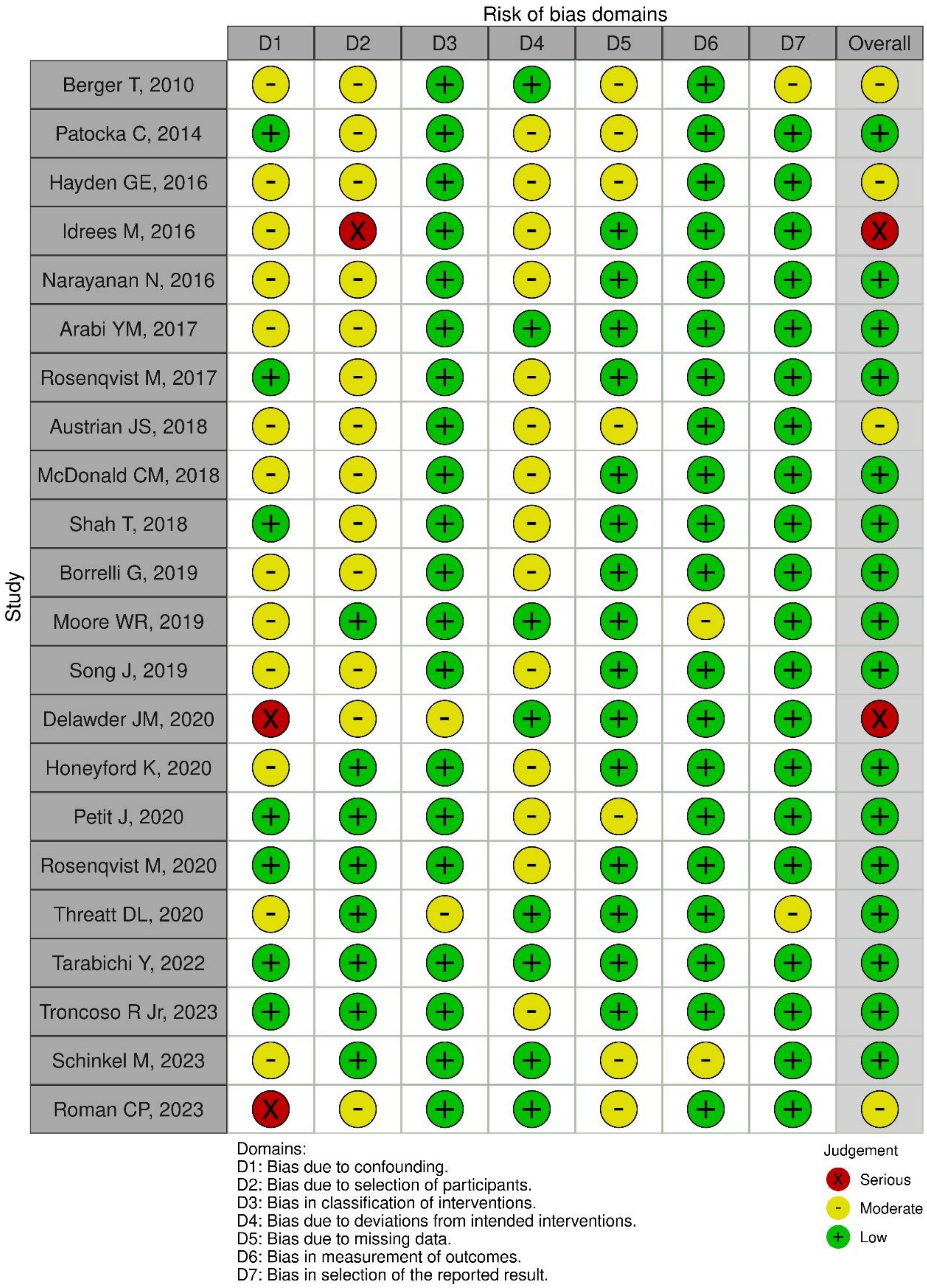

© 2024 Kim H et al. *JAMA Network Open.*

eFigure 5. Funnel Plot Analysis of Publication Bias for Association of Alert Systems in the Emergency Department with Patient Mortality

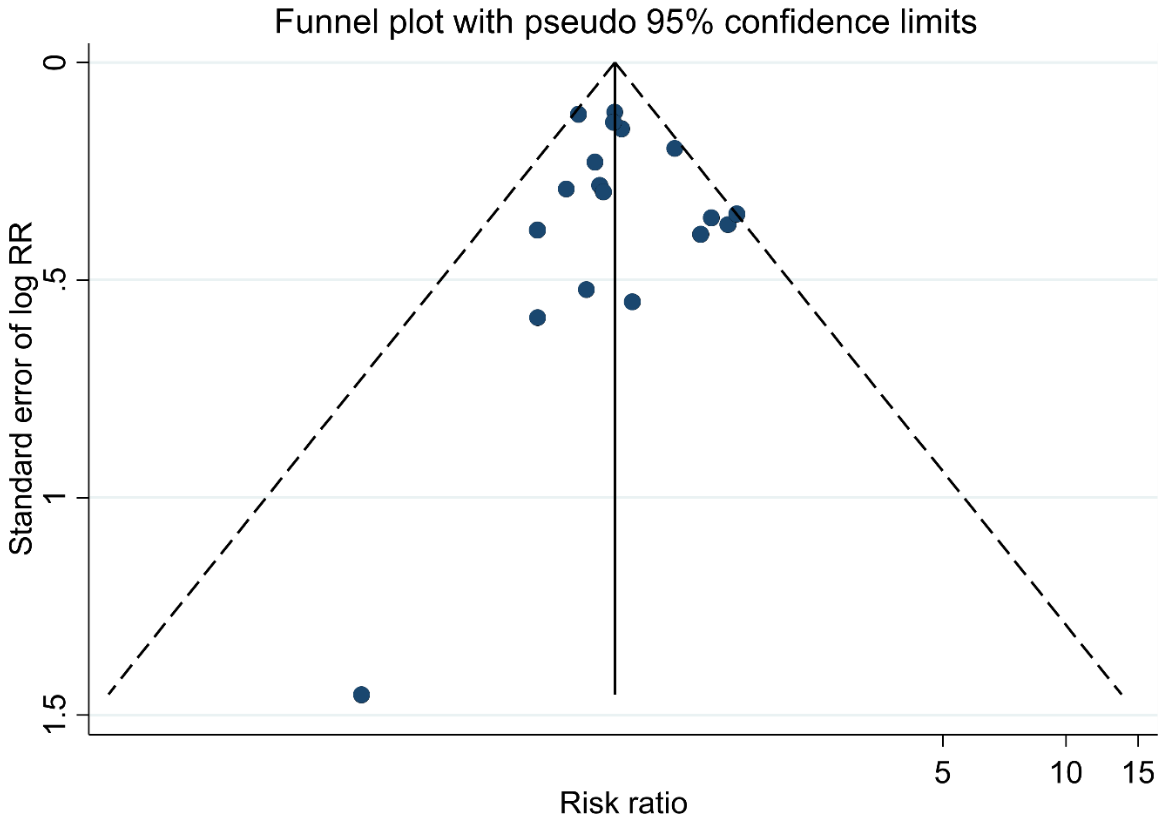

Egger's regression test did not detect significant publication bias (*p* = .53).

© 2024 Kim H et al. *JAMA Network Open.*
