# Supplementary material for: Sepsis Alert Systems, Mortality, and Adherence in Emergency Departments: A Systematic Review and Meta-Analysis
Source: JAMA Netw Open. 2024 Jul 22;7(7):e2422823. doi: 10.1001/jamanetworkopen.2024.22823 (PMC11265133; doi:10.1001/jamanetworkopen.2024.22823)
Supplement: Supplement 3. — Data Sharing Statement [file jamanetwopen-e2422823-s003.pdf]

Data Sharing Statement

Kim. Sepsis Alert Systems, Mortality, and Adherence in Emergency Departments. *JAMA Netw Open*. Published July 22, 2024. doi:10.1001/jamanetworkopen.2024.22823

Data
Data available: Yes
Data types: Data (not involving human participants)
How to access data: The data that support the findings of this study are available on request from the corresponding author.
When available: With publication

Supporting Documents
Document types: None

Additional Information
Who can access the data: Anyone who has a reasonable request
Types of analyses: Meta-analysis
Mechanisms of data availability: After approval of a proposal
